# Antibiotics-Induced Gut Microbiome Dysbiosis Affects Susceptibility to Minus Lens-Induced Myopia in Mice

**DOI:** 10.1167/iovs.66.6.76

**Published:** 2025-06-25

**Authors:** Shin-ichi Ikeda, Deokho Lee, Junhan Chen, Shinji Fukuda, Kazuno Negishi, Kazuo Tsubota, Toshihide Kurihara

**Affiliations:** 1 Laboratory of Photobiology, Keio University School of Medicine, Shinjuku-ku, Tokyo, Japan; 2 Department of Ophthalmology, Keio University School of Medicine, Shinjuku-ku, Tokyo, Japan; 3 Institute for Advanced Biosciences, Keio University, Kakuganji, Tsuruoka, Yamagata, Japan; 4 Gut Environmental Design Group, Kanagawa Institute of Industrial Science and Technology, Kawasaki-ku, Kawasaki, Kanagawa, Japan; 5 Transborder Medical Research Center, University of Tsukuba, Tsukuba, Ibaraki, Japan; 6 Laboratory for Regenerative Microbiology, Juntendo University Graduate School of Medicine, Bunkyo-ku, Tokyo, Japan; 7 Tsubota Laboratory, Inc., Shinjuku-ku, Tokyo, Japan

**Keywords:** myopia, myopia susceptibility, gut microbiome, clostridiaceae

## Abstract

**Purpose:**

The prevalence of myopia has increased worldwide in recent decades, shifting the focus in research from genetic to environmental factors. The roles of diet in the development of myopia may be directly associated with gut microbiota composition. Therefore this study evaluated the effects of antibiotic-induced gut dysbiosis on the development of negative lens-induced myopia.

**Methods:**

We administered several antibiotics (ampicillin, vancomycin, neomycin, or a mixture) to induce gut dysbiosis in male C57BL/6J mice with negative lens-induced myopia. Gut microbiome profiles were analyzed by 16 S rRNA gene sequencing.

**Results:**

Mice administered vancomycin, neomycin, and a mixture of three antibiotics exhibited resistance to lens-induced myopia, unlike control or ampicillin-administered mice. Further analyses revealed no specific trend in the gut microbiota composition and diversity related to myopia resistance, except for an increase in the abundance of Clostridiaceae.

**Conclusions:**

These findings demonstrate the potential role of the gut microbiome, particularly Clostridiaceae family, in myopia susceptibility. This study offers new insights into the preventive strategies and therapeutic interventions to mitigate myopia development.

The global prevalence of myopia (nearsightedness) has increased markedly in recent decades, especially among children and young adults.^1^^,^^2^ Historically, myopia was primarily considered a genetic disorder attributed to familial predisposition.^3^^–^^6^ However, the worldwide surge in myopia cases across age groups has challenged this hypothesis and underscored the potential influence of environmental factors on the development and progression of myopia. Lifestyle changes, such as increased screen time, reduced outdoor activities, and prolonged near-work tasks, have been implicated in the myopia epidemic.^7^^–^^9^ Outdoor activity is the most well-known environmental factor associated with the suppression of myopia progression, and we previously reported that light at wavelengths of 360–400 nm (violet light), such as that contained in natural sunlight, has a myopia-suppressing effect.^10^^–^^12^ Recent research suggested a possible link between dietary habits and myopia onset. An observational study reported that westernized dietary habits are associated with longer axial eye lengths in Japanese children aged 8–9 years.^13^ An epidemiological cross-sectional study in France showed that refined carbohydrate intake was associated with the development of myopia in females.^14^ Furthermore, several food-derived factors including lactoferrin, omega-3 polyunsaturated fatty acids, and crocetin have been shown to suppress myopia development in animal models and humans.^15^^–^^19^ These results suggest that changes in dietary habits may promote myopia development, and that dietary intervention may be effective in myopia management.

Dietary habits have a profound impact on the composition and function of the gut microbiota.^20^^–^^23^ Our intestinal lumen is the place for a complex ecosystem inhabited by trillions of microorganisms, collectively referred to as the gut microbiome, which plays pivotal roles in digestion, immune function, and metabolic processes.^24^^,^^25^ Alterations in dietary patterns can have both beneficial and detrimental effects on the gut microbiome, subsequently influencing the pathophysiological conditions not only in the intestinal tract but also in remote organs such as the brain, heart, liver, and eyes.^23^^,^^24^^,^^26^^–^^32^ Diet-derived factors with myopia-suppressive effects are known to affect the gut microbiome. Lactoferrin improved dry eye disease by modifying the gut microbiome and increasing short-chain fatty acid production.^33^ Omega-3 polyunsaturated fatty acid supplementation for eight weeks resulted in a reversible increase in the abundance of *Bifidobacterium* and *Lactobacillus* in human and mouse intestines.^34^^,^^35^ However, it is unclear whether diet-induced changes in the gut microbiome have any association with the onset and development of myopia. We hypothesized that changes in the gut microbiota may affect individual susceptibility to myopia. In the present study, we aimed to evaluate the above hypothesis in mice wearing negative lenses and presenting with gut dysbiosis caused by the administration of several antibiotics.

## Methods

### Mice

All animal experiments in this study were approved by the Animal Experimental Committee of Keio University (permit number: 16017) and adhered to the Institutional Guidelines on Animal Experimentation at Keio University, the ARVO Statement for the Use of Animals in Ophthalmic and Vision Research, and Animal Research: Reporting of In Vivo Experiments (ARRIVE) Guidelines for the Use of Animals in Research. Five to six male C57BL/6J mice (three weeks old; CLEA Japan, Yokohama, Japan) were housed in a cage under controlled conditions (12-hour light/dark cycle, lights switched at 8:00 AM/PM; 23°C ± 3°C) with free access to standard chow and water.

### Antibiotics Administration

**Figure 1.**
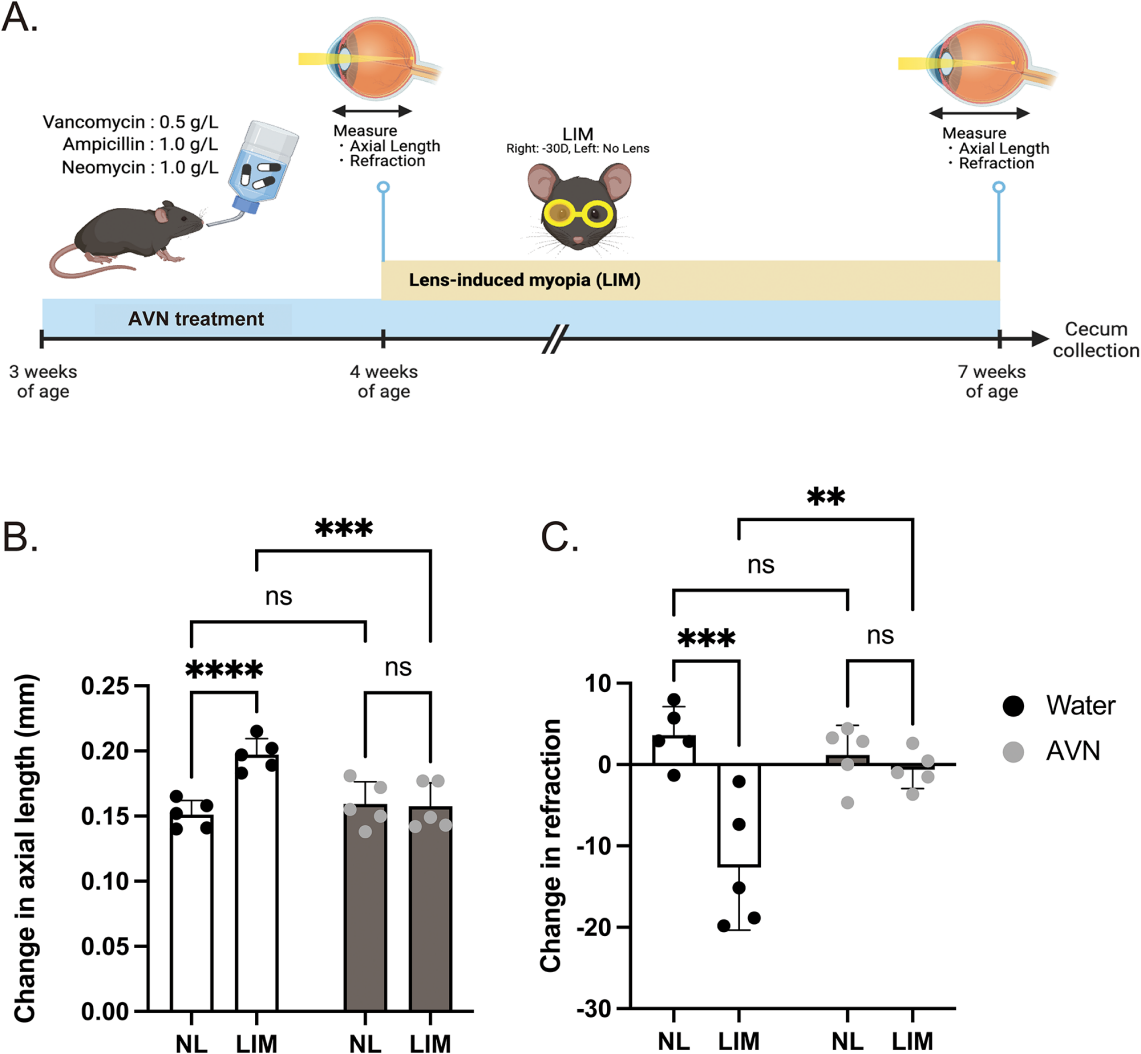
Antibiotics-induced dysbiosis affects lens-induced myopia (LIM) in mice. (**A**) Timeline for administering antibiotics (AVN) and inducing myopia. (**B**) Effects of AVN administration on changes in axial length after three weeks of wearing minus lens in male C57BL/6J mouse (*n* = 5 per group). (**C**) Effects of AVN administration on myopic shift in refraction after three weeks of wearing minus lens in male C57BL/6J mice (*n* = 5 per group). ** *P* < 0.01, *** *P* < 0.001, **** *P* < 0.0001 analyzed using two-way ANOVA with Fisher's LSD. The experiments are representative of three times independent replicates to confirm reproducibility.

To establish gut dysbiosis, mice (*n* = 5–6, respectively) were given sterilized water containing ampicillin (Amp, 1 g/L; Tokyo Chemical Industry, Tokyo, Japan), neomycin (Neo, 1 g/L; Tokyo Chemical Industry), and vancomycin (Van, 0.5 g/L; Chem-Impex International, Wood Dale, IL, USA) together (AVN) or separately for four weeks (from three to seven weeks old) (Fig. 1A). Control mice (*n* = 5–6) were administered sterile water for four weeks (Fig. 1A).

### Lens-Induced Myopia and Ocular Parameter Measurements

Lens-induced myopia (LIM) was performed at four to seven weeks of age, in parallel with antibiotics treatment, as previously reported.^17^ Briefly, the mice were treated with a mydriatic, and axial length and refraction were measured using spectral domain optical coherence tomography (Envisu R4310; Leica, Wetzlar, Germany) and refractometry (Steinberis Transfer Center, Tübingen, Germany). The skull was exposed and fixed with a special device for fitting frames over the eyes. A frame with a −30 diopter (D) lens was placed on the right eye, and a frame without a lens (as a control) was placed on the left eye.

### Gut Microbiome Analysis

After four weeks of antibiotic administration, the mice were placed in autoclaved cages individually and defecate naturally. The first four to five fecal pellets per mouse were collected using sterile tweezers. Fecal DNA extraction, 16S rRNA gene sequencing of the cecal microbial DNA, and 16S rRNA gene sequence analysis were performed using the Quantitative Insights into Microbial Ecology pipeline (version 1.9.1) as previously described.^36^ The log-fold change (logFC) and *P* value (Student's *t*-test) were calculated for the fecal samples (six feces/tube) of each seven-week-old mouse group (six samples/group) using R software (version 4.3.1; The R Foundation for Statistical Computing, Vienna, Austria). The groups were categorized as follows: (1) AVN-treated versus control; (2) Van-treated versus control; (3) Amp-treated versus control; and (4) Neo-treated versus control. A scatter plot and heatmap were used to compare the logFC of altered microbiota during myopia progression in the different groups.

### Short-Chain Fatty Acids (SCFAs) ELISA

After four weeks of antibiotic administration (Amp, Van, Neo or Water as a control) with LIM, mice were anesthetized, and blood samples were taken from the inferior vena cava using a heparinized syringe and needles. Blood samples were spun in a centrifuge, and the supernatant (plasma) was collected. SCFAs ELISA was performed using the Mouse Short Chain Fatty Acid (ScFA) ELISA kit (antibodies.com, Cambridge, UK) according to the manufacturer's protocol.

### Statistical Analysis and Reproducibility

Sample size calculations were performed using G*Power software version 3.1.9. Differences between the control and experimental groups were compared using analysis of variance with the least significant difference (LSD) post hoc or Tukey–Kramer tests to calculate statistical significance (GraphPad Prism software, version 8.0; GraphPad Software, San Diego, CA, USA). All experiments are representative of at least two independent replicates to confirm reproducibility.

## Results

### Antibiotics-Induced Gut Dysbiosis Attenuates Minus Lens–Induced Myopia Development in Mice

To determine whether the gut microbiome is associated with myopia development, we provided mice with drinking water containing a mixture of vancomycin (Van, 0.5 g/L), ampicillin (Amp, 1 g/L), and neomycin (Neo, 1 g/L) to disturb the gut microbiome over four weeks (from three to seven weeks of the mouse age), performed LIM for three weeks (from four to seven weeks of the age), and then compared the degree of myopia between the AVN-treated and vehicle-tread (sterilized water: Water) mice. Compared with no-lens (NL) control eyes, LIM-induced eyes showed a much longer axial elongation (Fig. 1B) with a myopic shift in refraction (Fig. 1C) in the Water group. However, both refraction and axial length were comparable between AVN-treated NL and LIM eyes (Fig. 1B and 1C).

### Antibiotics Have Different Suppressive Effects on Lens-Induced Myopia in Mice

**Figure 2.**
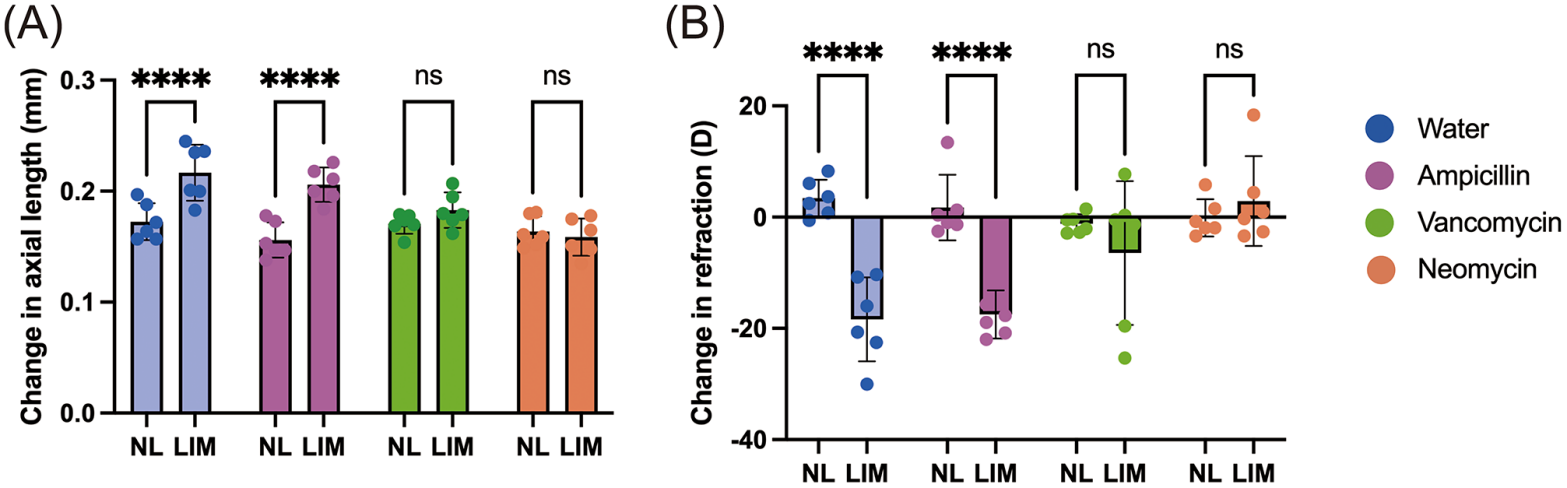
Neomycin- and vancomycin-administrated mice show resistance to myopia induction with minus lens. (**A**) Effects of vancomycin, ampicillin, or neomycin administration on changes in axial length after three weeks of wearing minus lens in male C57BL/6J mouse (*n* = 6 per group). (**B**) Effects of vancomycin, ampicillin, or neomycin administration on changes in refraction after three weeks of wearing minus lens in male C57BL/6J mouse (*n* = 6 per group). *** *P* < 0.001, **** *P* < 0.0001 analyzed using two-way ANOVA with Fisher's LSD. The experiments are representative of twice independent replicates to confirm reproducibility.

AVN administration abolished the development of LIM in mice. To further elucidate the association between myopia development and the gut microbiome, we evaluated the effect of each antibiotic in LIM mice. A separate antibiotic [Van, Amp, Neo, or water (control)] was administered and LIM was induced using the same protocol as in the AVN-administration experiment (Fig. 1A). Changes in axial length and refraction were similar between the Water and Amp groups, and both presented myopia (Figs. 2A, 2B). However, NL and LIM eyes subjected to Van or Neo treatment showed little change in axial elongation and refraction (Figs. 2A, 2B).

### Antibiotics-Induced Comprehensive Changes in the Gut Microbiota Do Not Correlate With Myopia Suppression in Mice

**Figure 3.**
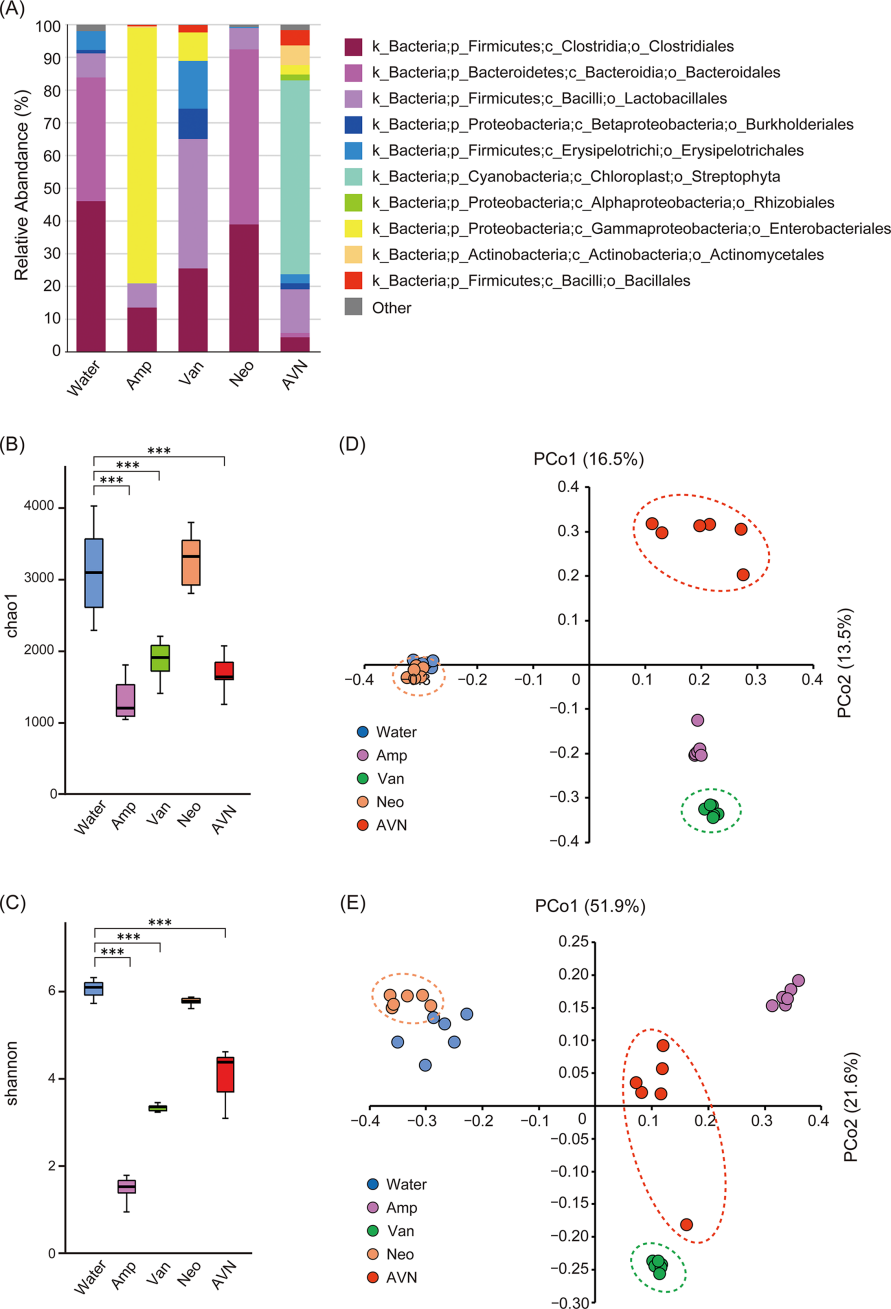
Alterations in gut microbiome profiles and potential associations with myopia suppression in antibiotics-administrated mice. (**A**) Relative abundance of top-10 most abundant genera (average abundance in all samples) in water (control) or antibiotic-treated groups (Van, Amp, Neo, AVN). (**B**, **C**) Chao1 (**B**) and Shannon (**C**) indices. The lower and upper limits of each box define the 25th and 75th percentiles, whiskers are the minimum and maximum values, and the median is presented by *black lines*. *** *P* < 0.001, analyzed using Tukey–Kramer test. (**D**, **E**) Unweighted (**D**) and weighted (**E**) UniFrac PCoA plots. *Dotted lines* delineate samples showing resistance to the progression of negative lens-induced myopia. All groups comprised six samples. The experiments are representative of three times independent replicates to confirm reproducibility.

We hypothesized that certain gut microbiome profiles may exert tolerance to the development of myopia. To evaluate this hypothesis, the gut microbiome composition was evaluated using 16S rRNA gene sequencing (see the Methods section). The relative abundance of microbial orders in Figure 3A demonstrated that the administration of antibiotics affected the gut microbiome composition in the treatment groups compared with the Water group.

The alpha (“within-sample”) diversity Chao1 and Shannon indexes were employed to estimate the bacterial richness and evenness in the gut microbiome among the groups. The total bacterial richness estimated by the Chao1 index was significantly decreased in the Amp, Van and AVN groups, but not in the Neo group, compared to the Water group (Fig. 3B). The Shannon index, which reflects both the richness and evenness of the microbiome, showed the same trend as the Chao1 index (Figs. 3B, 3C). However, there were no common trends in diversity between control and antibiotics with no suppressive effects on myopia progression(Water and Amp) or antibiotics with suppressive effects on myopia progression (Van, Neo, and AVN) groups. These results suggested that the antibiotics-induced perturbation in gut microbiome diversity may not directly contribute to the suppression of myopia development in the LIM mouse model.

Beta diversity (indicating the relative differences in community composition between groups) was assessed using unweighted and weighted UniFrac principal coordinate analysis (PCoA) to represent the degree of variation among the Water, Amp, Van, Neo, and AVN groups (Figs. 3D, 3E). The five groups were separated into distinct clusters, implying that distinct microbial community structures may exist for each antibiotic treatment. There was a large distance between the two myopia-sensitive groups (Water and Amp) and tolerant groups (Van, Neo, and AVN), while the close distance between the Water (myopia-prone) and Neo (myopia-resistant) groups suggested that the composition of the gut microbiota may not be closely involved in the development of myopia.

### Clostridiaceae Abundance Correlates With Myopia Status Under Specific Antibiotic Administration

**Figure 4.**
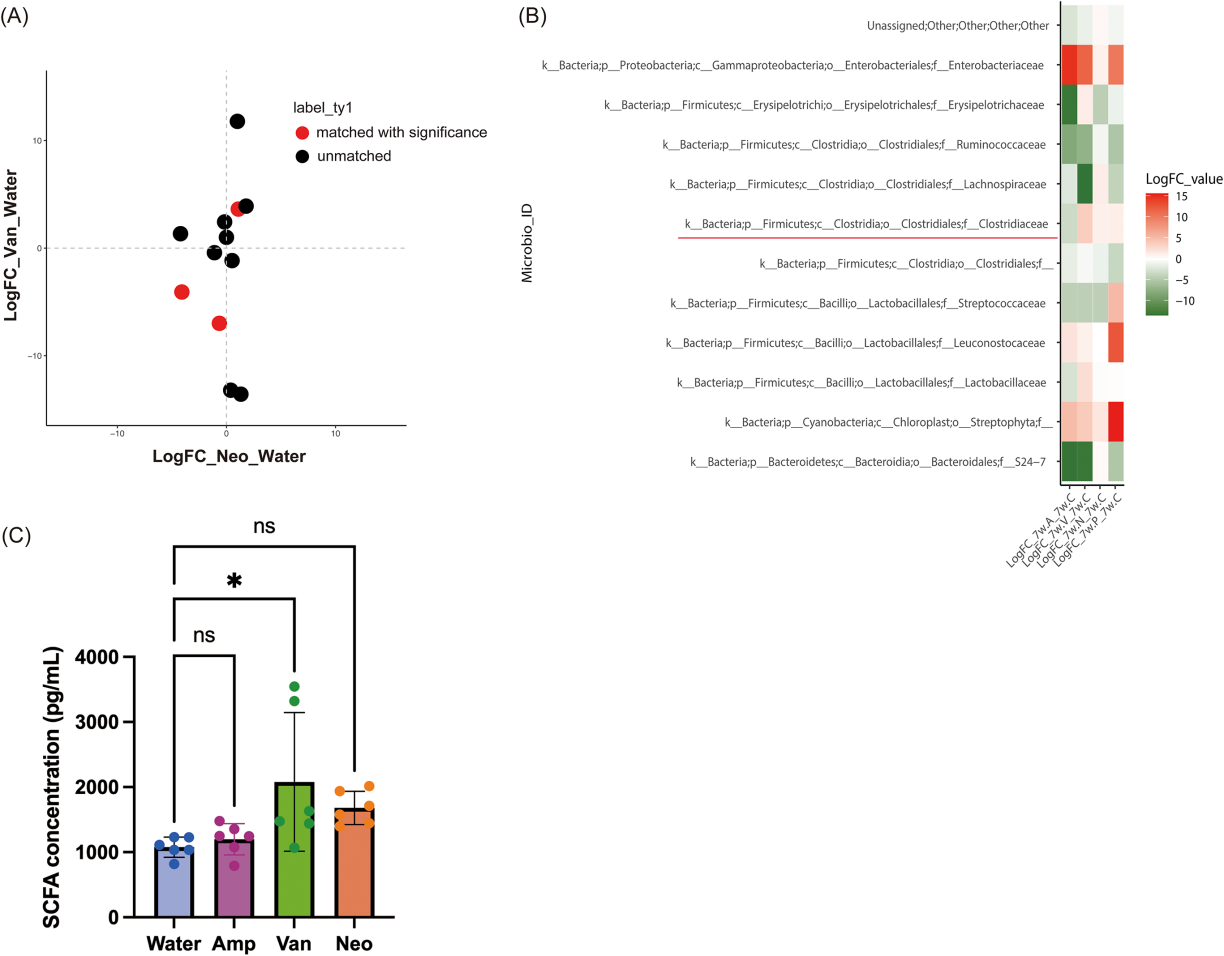
Identification of specific gut microbes associated with myopia suppression. (**A**) Scatter plot of logFC calculated in the neomycin-treated versus control (x-axis) and vancomycin-treated versus control group (y-axis). Similar trends in microbiota abundance observed in the neomycin- and vancomycin-treated groups are colored red (matched with significance): k__Bacteria;p__Firmicutes;c__Clostridia;o__Clostridiales;f__Ruminococcaceae, k__Bacteria;p__Firmicutes;c__Clostridia;o__Clostridiales;f__Clostridiaceae, and k__Bacteria;p__Firmicutes;c__Bacilli;o__Lactobacillales;f__Streptococcaceae. The others are colored *black* (unmatched). (**B**) Heatmap demonstrating logFC data in all groups: (1) AVN -treated versus control, (2) Vancomycin-treated versus control, (3) Ampicillin-treated versus control, (4) Neomycin-treated versus control. Only Clostridiaceae was matched with the increasing trends in the neomycin-treated, vancomycin-treated, and AVN-treated groups, and decreasing trend in the ampicillin-treated group. LogFC values were colored from *green* (lowest, negative) to *red* (highest, positive), with *white* indicating the midpoint (0). All groups consisted of six samples. (**C**) Plasma SCFA levels in control or antibiotic-administered group (Amp, Van, Neo) measured by ELISA. *P < 0.05; ns, not significant analyzed using one-way ANOVA with the Tukey post hoc test. These experiments are representative of two times independent replicates to confirm reproducibility.

Because the microbiota diversity profiles (alpha- and beta-diversity) did not correlate with the trends in myopia development between antibiotic treatments, we investigated the involvement of particular bacteria. First, we identified the microorganisms showing the same changes in abundance following the administration of Van and Neo (Fig. 4A). Among the 12 categories, three (k__Bacteria;p__Firmicutes;c__Clostridia;o__Clostridiales;f__Ruminococcaceae, k__Bacteria;p__Firmicutes;c__Clostridia;o__Clostridiales;f__Clostridiaceae, and k__Bacteria;p__Firmicutes;c__Bacilli;o__Lactobacillales;f__Streptococcaceae) were statistically significant. The abundance of Clostridiaceae increased in Neo- or Van-treated samples, whereas that of Ruminococcaceae and Streptococcaceae decreased. Because Amp did not exert suppressive effects on myopia progression (Fig. 2), both Ruminococcaceae and Streptococcaceae were excluded since they showed similar trends to those in the Neo- or Van-treated groups (Fig. 4B, fourth and eighth rows from the top). Clostridiaceae showed a consistent increasing tendency not only in the Neo- and Van-treated groups but also in the AVN-treated group. The abundance of Clostridiaceae tended to decrease in the Amp-treated group.

### Administration of Antibiotics With Suppressive Effects on Myopia Progression Tended to Increase Plasma Short-Chain Fatty Acids in Mice

The gut microbiome affects not only the gut but also other organs by producing a range of metabolites such as SCFAs, which can enter the blood stream and travel to distant organs. SCFAs have anti-inflammatory effects and Clostoridium is a bacteria producing SCFAs.^37^^–^^40^ Because SCFAs also affects ocular inflammatory responses and oral administration of anti-inflammatory substances such as crocetin and lactoferrin can suppress the myopia development,^17^^,^^19^^,^^33^^,^^41^ we inferred the SCFAs are associated with antibiotics-induced change of susceptibility to myopia in our experimental settings. After 4 weeks of antibiotic administration, the plasma of each mouse was corrected and the levels of SCFAs were evaluated by ELISA. Compared to control group, Van group showed the higher levels of SCFAs in the plasma (Fig. 4C). The Neo group tended to have higher plasma SCFAs than the control group but not significant. These data suggest that an increase in plasma SCFA levels is associated with susceptibility to the development of myopia.

## Discussion

In this study, we found that antibiotics-induced gut dysbiosis affected the susceptibility to LIM in mice, and the abundance of the bacterial family Clostridiaceae in the gut microbiome was correlated with the susceptibility to myopia. Myopia is a multifactorial disorder thought to be caused by the interaction between genetic and environmental factors.^42^ The involvement of specific environmental factors such as the outdoor activity and near work, have been extensively studied^7^^–^^9^ and have identified several mechanisms underlying the development and progression of myopia.^11^^,^^12^. However, the role of the gut microbiome in the development of myopia remains unclear. Dietary habits have been correlated with myopia prevalence in some cohorts^13^^,^^14^ and animal studies have shown that myopia is suppressed by diet-based interventions.^15^^,^^17^^–^^19^ Therefore we hypothesized that changes in the gut microbiome affect the susceptibility to myopia development. Accordingly, mice were subjected to antibiotic administration to induce gut microbiome dysbiosis and negative lens treatment to induce myopia. Mice with antibiotics (Van, Neo and AVN)-induced dysbiosis showed lower susceptibility to LIM than control mice with a normal gut microbiome. However, mice administered with Amp showed normal development of myopia. These results suggested that gut microbiome structure determines the susceptibility to myopia development.

AVNM (an antibiotic cocktail containing ampicillin, vancomycin, neomycin, and metronidazole) is commonly used to deplete the gut microbiome in mice. However, we only used three antibiotics, excluding metronidazole, which may affect the drinking habits of mice and led to dehydration in our experimental setting. Further, we used three-week-old mice, as juvenile mice are more susceptible to experimental myopia induction than older one.^43^^,^^44^ Unfortunately, juvenile mice may be less tolerant of the bitterness of metronidazole than adult mice and showed aversive responses in the present study. Bitter aversion is an innate response found in mammals.^45^^,^^46^ With experience, the stimulus becomes more acceptable, and does not necessarily cause aversion.^47^^,^^48^ Our preliminary study provided sufficient evidence to exclude metronidazole and reduce the undesired outcomes induced by dehydration, which should be addressed in future studies. We confirmed that AVN caused enough disturbance in the gut microbiome to study its effect on the progression of myopia.

Ampicillin is a broad-spectrum beta-lactam antibiotic that penetrates both Gram-positive and Gram-negative bacteria.^49^ Vancomycin is a broad-spectrum glycopeptide with activity against Gram-positive bacteria including methicillin-resistant *Staphylococcus aureus* and drug-resistant *Clostridium difficile.*^50^ Neomycin is an aminoglycosidic antibiotic effective against Gram-negative proteobacteria.^51^ All antibiotics are known to alter the gut microbiome in mice. Ampicillin or vancomycin administration results in a decrease in *Bacteroidetes* and increase in *Proteobacteria* abundance,^52^^–^^55^ and neomycin treatment favors *Bacteroidetes* dominance.^56^^,^^57^ The overall changes in the gut microbiota observed in this study are consistent with those reported in previous studies. The decrease in Chao1 and Shannon indices, indicators of alpha diversity, in the Amp-, Van-, and AVN-treated groups (except Neo) was also comparable to previous findings,^57^ indicating the reproducibility of the AVN antibiotic treatment piece.

In this study, the water and Amp groups were LIM-sensitive, whereas the Van, Neo, and AVN groups were LIM-resistant, despite also wearing negative lenses. Therefore we hypothesized that antibiotic-induced differences in the gut microbiome affects resistance to myopic stimuli. The 16S rRNA gene sequencing analysis and PCoA of the gut microbiome samples revealed no specific trend in either the myopia-sensitive groups or myopia-resistant groups. Therefore we focused on the changes in individual gut microbiota and found that the relative abundance of Clostridiaceae was notably greater in the myopia-resistant groups (Van, Neo, and AVN) than in the myopia-sensitive group (Amp). The Clostridiaceae family includes several bacteria that have diverse harmful and beneficial effects on human health. *Clostridium*, first isolated in the 19^th^ century by Louis Pasteur, is a genus of anaerobic, Gram-positive, rod-shaped, spore-forming bacteria found in diverse environments such as soil, water, and the alimentary tracts of humans and other animals. These bacteria perform essential metabolic functions by converting starch, proteins, and purines into organic acids, alcohols, CO_2_, and hydrogen, which play essential roles in various ecosystems.^58^ *Clostridium* spp., particularly *C. butyricum*, are beneficial for human health because they produce SCFAs that support intestinal homeostasis,^59^ enhance immune function,^60^^–^^62^ and alleviate inflammation.^63^ Certain strains of *Clostridium* are used as probiotics^62^ to promote gut health, prevent infections,^64^ and potentially aid in the treatment of various inflammation-related diseases, such as inflammatory bowel disease,^65^ diabetes,^61^ and cancer.^66^^,^^67^ Although more detailed, species-level identification is needed in future studies, probiotics may be effective in preventing myopia in children because some *Clostridium* species suppresses inflammation, which may counteract the onset and development of myopia.^68^^,^^69^ The intake of immunomodulatory and immunosuppressive substances inhibits myopia progression.^15^^,^^17^ Similar to our findings on the intestinal Firmicutes family Clostridiaceae, the induction of myopia resulted in a decrease in the relative abundance of Firmicutes in C57BL/6J mice.^70^ Based on our previous findings, specific Clostridiaceae species may be associated with myopia development and progression, requiring further in-depth research.

Clostridium is known as one of the SCFAs-producing bacteria. SCFAs play a crucial role in various biological process, such as maintaining gut homeostasis, regulating lipid and glucose metabolism and modulating the immune responses thorough their anti-inflammatory properties.^71^ The inflammatory response is implicated in the onset and progression of myopia, as evidenced by its association with inflammatory diseases such as type 1 diabetes, uveitis, and systemic lupus erythematosus in humans, and its induction in animal models of myopia.^68^ Furthermore, oral administration of anti-inflammatory substances can suppress the myopia development.^17^^,^^19^ This study found that the Van and Neo groups exhibited a higher population of Clostridiaceae in the gut and elevated levels of SCFAs in plasma, suggesting that SCFAs produced by Clostridiaceae may contribute to myopia suppression via their anti-inflammatory effects. However, we have not confirmed whether increased SCFAs inhibit myopia, nor have we identified which SCFAs are associated with myopia, future research is needed to uncover these points.

Several limitations exist in the present study. First, we conducted all our experiments on mice, and its relevance to the human myopia needs to be determined. Also, the exclusive use of male C57BL/6J mice restricts the applicability of findings to other sexes and strains. Second, our study relied on 16S rRNA gene sequencing, which provides limited resolution at the species level. Thirdly, while our study identified a correlation between Clostridiaceae abundance, SCFAs level and myopia suppression, it did not elucidate the causal relationships. To overcome second and third limitations, meta transcriptmics analysis that can help identify the biochemical functions of a complex microbial community and how it is impacted by interventions and metabolome analysis which further provide possible mechanisms how Clostridiaceae mediate their beneficial effect to prevent myopia should be incorporated in future research to gain a more comprehensive and mechanistic understanding of the gut-eye axis in myopia development.

In conclusion, our results revealed a strong correlation between antibiotic-induced dysbiosis and myopia susceptibility, highlighting the pivotal role of the gut microbiome in modulating the of myopia—an increasingly common visual disorder. The abundance of specific bacteria, particularly of the family Clostridiaceae, emerged as a potential environmental factor associated with the susceptibility to myopia in juvenile mice. These findings not only underscore the potential impact of dietary habits on myopia prevalence but also highlight the potential contributions of a novel environmental factor—the gut microbiome—on the development of myopia through its intricate interplay with genetic and environmental factors. These findings open new research avenues on the intricate mechanisms by which changes in the gut microbiome composition may influence susceptibility to myopia. Our study highlights the potential of therapeutic interventions that modulate the gut microbiome for mitigating the development of myopia and offers exciting prospects for future therapeutic strategies.
